# Characterization of Retinal Ganglion Cell and Optic Nerve Phenotypes Caused by Sustained Intracranial Pressure Elevation in Mice

**DOI:** 10.1038/s41598-018-21254-8

**Published:** 2018-02-12

**Authors:** Guofu Shen, Schuyler Link, Sandeep Kumar, Derek M. Nusbaum, Dennis Y. Tse, Yingbin Fu, Samuel M. Wu, Benjamin J. Frankfort

**Affiliations:** 1 Department of Ophthalmology, Baylor College of Medicine, Houston, TX USA; 2 Department of Neuroscience, Baylor College of Medicine, Houston, TX USA; 3 School of Optometry, The Hong Kong Polytechnic University, Hong Kong, Hong Kong

**Keywords:** Retina, Neurological disorders

## Abstract

Elevated intracranial pressure (ICP) can result in multiple neurologic sequelae including vision loss. Inducible models of ICP elevation are lacking in model organisms, which limits our understanding of the mechanism by which increased ICP impacts the visual system. We adapted a mouse model for the sustained elevation of ICP and tested the hypothesis that elevated ICP impacts the optic nerve and retinal ganglion cells (RGCs). ICP was elevated and maintained for 2 weeks, and resulted in multiple anatomic changes that are consistent with human disease including papilledema, loss of physiologic cupping, and engorgement of the optic nerve head. Elevated ICP caused a loss of RGC somas in the retina and RGC axons within the optic nerve, as well as a reduction in both RGC electrical function and contrast sensitivity. Elevated ICP also caused increased hypoxia-inducible factor (HIF)-1 alpha expression in the ganglion cell layer. These experiments confirm that sustained ICP elevation can be achieved in mice and causes phenotypes that preferentially impact RGCs and are similar to those seen in human disease. With this model, it is possible to model human diseases of elevated ICP such as Idiopathic Intracranial Hypertension and Spaceflight Associated Neuro-ocular Syndrome.

## Introduction

Vision loss is a feared complication of elevated intracranial pressure (ICP), and visual changes can be rapid and profound at extreme levels of ICP, but may be more chronic and insidious at moderate levels of ICP. Moderate levels of ICP elevation are associated with number of clinical entities, including Idiopathic Intracranial Hypertension (IIH) and Spaceflight Associated Neuro-ocular Syndrome (SANS; formerly Vision Impairment and Intracranial Pressure [VIIP] Syndrome), and these diseases are associated with chronic visual changes that are at times progressive^1–3^. The site of injury during ICP elevation is the optic nerve (consisting of retinal ganglion cell [RGC] axons) and the optic nerve head. Not surprisingly, there is evidence of optic nerve and RGC loss in IIH^4–7^. Furthermore, the balance between ICP and intraocular pressure (IOP), which together provide a pressure gradient across the optic nerve head, contributes not only to diseases of elevated ICP, but to diseases of elevated IOP, such as glaucoma^8–11^.

Currently, the precise mechanism of ICP-related vision loss is unclear, although a wide array of human and animal data suggest that papilledema is the critical anatomic prerequisite^12–14^. Within this context, neuronal ischemia secondary to mechanical trauma and alterations to axoplasmic flow and optic nerve head vasculature is thought to be a key component^12,15^. To better understand these processes, there has been considerable interest in identifying animal models to assist in the study of IIH, papilledema, and elevated ICP^16–27^. Unfortunately, many of the existing disease models either do not allow for sustained ICP elevation in living animals, do not allow for customizable ICP elevation to levels at the discretion of the investigators, or cannot be performed in genetically tractable organisms such as mice. Therefore, our ability to study the mechanisms which underlie phenotypes related to increased ICP remains limited.

We have previously reported a novel model for the experimental modulation and measurement of ICP in living, awake, active mice, and confirmed that extreme ICP elevation to levels greater than 30 mmHg (three times baseline) causes both RGC and optic nerve axon loss^27^. However, neither this model nor others has been used to study the sustained ICP elevations to the more moderate levels that are characteristic of chronic human disease. In this manuscript, we report modifications to this model which allow for sustained, moderate ICP increases and test the hypothesis that these increases cause optic nerve, RGC axon, and RGC soma degeneration with concomitant physiologic abnormalities. To do so, we performed an extensive survey of visual system abnormalities following ICP elevation including anatomic, histologic, ischemic, electrophysiologic, and behavioral phenotypes. We demonstrate that elevated ICP primarily impacts the optic nerve and the RGC layer of the retina (ganglion cell layer, GCL). Importantly, these properties are similar to the key ocular findings seen in human diseases of elevated ICP and suggest that this model can be used for the detailed mechanistic study of ICP-related diseases in mice.

## Results

### Moderate ICP elevation can be sustained

**Figure 1:**
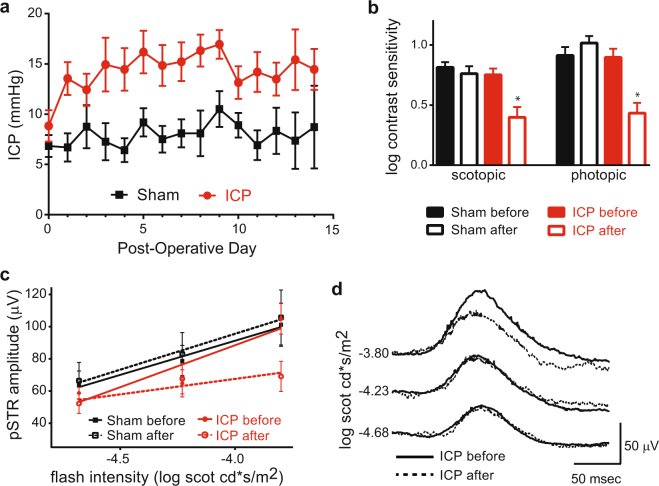
ICP elevation is stable and causes reduced behavioral contrast sensitivity and RGC electrical responses. (**a**) ICP readings for Sham (black) and ICP (red) animals plotted for the entire 2-week experiment. ICP was elevated on Post-Operative Day (POD) 1 and maintained at an increased level in the ICP group. ICP elevated animals had a significantly higher ICP from POD 1–14 when compared to POD 1–14 for the Sham group (ANOVA; p < 0.0001). N = 19 animals for Sham and N = 17 animals for ICP. Error bar = 1 SEM. (**b**) Log contrast sensitivity was measured initially on the day after probe implantation, just prior to experimental ICP elevation (Sham before and ICP before) and again after 2 weeks (Sham after and ICP after) under both scotopic (left) and photopic (right) conditions. A significant decrease in contrast sensitivity was observed after 2 weeks of ICP elevation under both scotopic and photopic conditions (asterisks; t test, p < 0.0001 for each). N = 12 eyes for Sham and N = 16 eyes for ICP. Error bar = 1 SEM. (**c**) Mean electroretinogram (ERG) pSTR amplitude plotted against the intensity of scotopic flash stimuli. The best-fit linear regression lines of pSTR amplitude growth are indicated for each condition (red and black solid and dotted lines). The magnitude of the linear regression slope of pSTR growth at the end of the 2-week experiment is reduced only in the setting of increased ICP (t test comparing ICP after to ICP before, p = 0.01; t test comparing Sham after to Sham before, p = 0.33). N = 26 eyes for Sham and N = 12 eyes for ICP. Error bar = 1 SEM. (**d**) Plot of average pSTR curves before (solid line) and after (dotted line) 2 weeks of ICP elevation (N = 12 eyes). There is a decreased pSTR amplitude with flash stimulus at the brightest tested scotopic intensity.

We have previously shown that ICP can be elevated to approximately three times baseline for one week, and that this elevation results in profound anatomic changes to the visual system^27^. However, this magnitude of ICP elevation is supra-physiologic and insufficient to model disease of elevated ICP such as IIH and SANS. Furthermore, the use of an albino CD1 mouse strain in this initial study precluded most functional evaluations of vision^28^. To overcome these limitations, we adapted our model to C57BL/6 J mice, despite their smaller size, and elevated ICP to a less severe level for a longer duration. Mice either underwent probe implantation surgery without infusion of artificial CSF (ACSF; Sham) or underwent probe implantation with infusion of ACSF (ICP). The average initial ICP was equivalent between the two groups (6.84 ± 1.04 mmHg and 8.84 ± 1.57 mmHg for Sham and ICP, respectively; t test, p = 0.304). ICP was then either increased (ICP) or maintained at baseline as a control (Sham) and measured for a duration of 2 weeks (Fig. 1a). The average daily post-operative ICP was stable at 7.85 ± 1.07 mmHg for the Sham group (t test, p = 0.503 compared to baseline measurement) and increased to 14.68 ± 1.64 mmHg for the ICP group (t test, p < 0.001 compared to baseline measurement). The IOP was measured throughout the study at specific intervals in both groups and found to be equivalent between the two groups (9.94 ± 0.21 mmHg and 9.99 ± 0.30 mmHg for Sham and ICP, respectively (t test; p = 0.906), suggesting that neither the model nor ICP elevation impacted IOP levels.

### Sustained ICP elevation reduces visual function and RGC electrical responses

Vision changes are a common feature of diseases of elevated ICP, and the severity of visual impairment and response to treatment can be monitored with tests of visual function^2,3,29^. In patients with other optic neuropathies, such as glaucoma, impairment of contrast sensitivity is among the earliest measurable changes^30–33^. Interestingly, this is also true for mice with experimental glaucoma, in whom scotopic and photopic behavioral contrast sensitivities are rapidly impacted^34,35^. As elevated ICP is expected to cause an optic neuropathy, we therefore hypothesized that behavioral contrast sensitivity in mice exposed to sustained ICP elevation would be diminished. We tested this by measuring the contrast-dependent optokinetic response (OKR) at baseline and at the conclusion of the 2-week experiment. After 2 weeks of elevation, the ICP group showed a marked reduction in both scotopic and photopic contrast sensitivity (Fig. 1b).

To determine if sustained ICP elevation primarily injured RGCs and their axons, we probed RGC electrical function with a whole field flash ERG, and focused on the positive scotopic threshold response (pSTR), which can reliably isolate RGC electrical function in normal and disease states^36–42^. Both Sham and ICP groups were tested at baseline and at the conclusion of the 2-week experiment. At baseline, for both groups (Sham before and ICP before) the pSTR displayed a characteristic increase in amplitude with increasing stimulus intensity (Fig. 1c). This amplitude growth (as indicated by the linear regression slope of the best fit lines) was preserved in the Sham group after 2 weeks but lost in the ICP group (Fig. 1c and d), suggesting a deficit in RGC electrical function. Corresponding analyses of the b-wave at stronger light intensities did not reveal any changes to amplitude growth with increasing light intensity (Supplemental Figure S1), suggesting that ICP-related electrical dysfunction is restricted to RGCs.

### Sustained ICP elevation impacts RGC and optic nerve anatomy

To further understand the impact of sustained ICP elevation on the retina and optic nerve, we performed an extensive anatomic analysis in both living and post-mortem experimental animals. Since the impact of elevated ICP is expected to be primarily on the optic nerve and optic nerve head, and RGC-related phenotypes have been identified in the setting of elevated ICP, we hypothesized that RGCs and their axons in the optic nerve would display anatomic evidence of damage, with relative or total sparing of the rest of the retina^27,42^.

**Figure 2:**
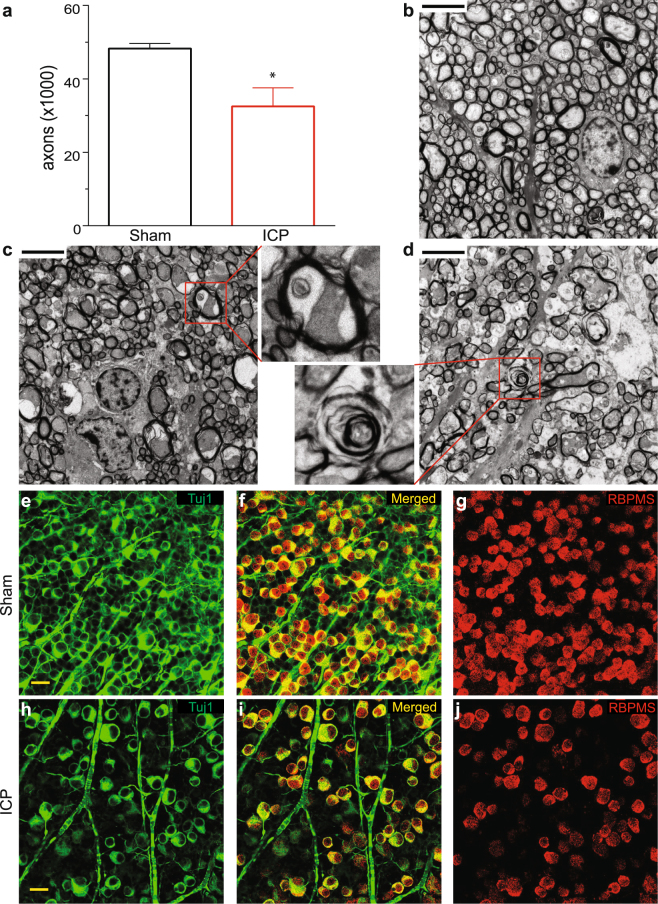
ICP elevation causes degeneration and loss of RGC axons and somas. (**a**) Axon counts for ICP (red) and Sham (black) groups after 2 weeks of ICP elevation. The number of axons per optic nerve is reduced after 2 weeks of ICP elevation (t test; p = 0.0097). N = 6 eyes for Sham and N = 5 eyes for ICP. Error bar = one SEM. (**b–d**). Representative optic nerve TEM images from Sham (**b**), moderate axon loss (**c**), and severe axon loss (**d**) after 2 weeks of ICP elevation. Note the relatively uniformly stained, small diameter axons in the Sham group (**b**) and different degrees of axon loss in (**c**) and (**d**). The upper inset indicates vacuolization inside the cytosol of an axon and lower inset indicates degeneration of an axolemma. Scale bar = 4 μm. 3000X magnification. (**e–j**). Representative confocal images from whole mount retinas stained for the RGC-specific markers Tuj1 (**e**/**f**,**h**/**i**) and RBPMS (**f**/**g**,**i**/**j**) after 2 weeks of either Sham or ICP elevation. Both RGC markers are reduced (Table 1). Scale bar = 20 μm. 40× magnification.

Sustained ICP elevation caused marked axonal loss, with a reduction from 48,267 ± 1,393 axons in the Sham group to 32,527 ± 5,054 in ICP group (Fig. 2a). As the standard error was higher in the elevated ICP group, we calculated the coefficient of variation (CV) between ten different regions (two central regions and eight random peripheral regions of each quadrant) of each optic nerve and found that the ICP elevation group had significant higher CV (17.0 ± 1.0) than the Sham group (12.1 ± 1.1), suggesting that axon loss did not occur evenly throughout the nerve. However, we were not able to detect any specific regional bias of optic nerve injury. We also found that while axonal degeneration is not seen in the Sham group (Fig. 2b), multiple stages of axonal degeneration are detected in the optic nerves exposed to elevated ICP, including swollen axons, empty myelin sheaths and myelin sheath degeneration (Fig. 2c,d). Other findings, such as enlarged non-neuronal nuclei and intra-axonal spaces were also observed, but it is unclear if these are direct effects of ICP elevation or secondary to the degradation of RGC axons. Interestingly, our previous study also found a range of optic nerve changes following ICP elevation, suggesting that inter-animal and intra-nerve variability may be consistent findings in this model^27^.

RGC somas were assessed in the ganglion cell layer (GCL) of retinal flat mounts using the RGC markers RBPMS and Tuj1^43,44^. As axon loss did not occur evenly throughout the nerve and there is conflicting evidence regarding the susceptibility of peripheral and central RGCs to damage at the optic nerve head in mice, we assessed RGC density in both central and peripheral regions with an established protocol^37^. RGC density was decreased with both markers in both central and peripheral retinal regions (Fig. 2e–j, Table 1). The difference in magnitude of loss between central and peripheral regions was not significant for either RBPMS or Tuj1 (t test; p > 0.05 for both). Other cells of the GCL were observed via counterstain with Topro3 and were not reduced by sustained ICP, suggesting that cell loss occurred only among RGCs (Supplemental Table S1).

**Table 1:** RGC counts.

	Sham	Elevated ICP	P
Tuj1 - central	4,475 ± 487	2,926 ± 277	0.009
Tuj1 - peripheral	4,306 ± 453	3,160 ± 254	0.030
RBPMS - central	3,439 ± 222	2,495 ± 179	0.006
RBPMS - peripheral	2,907 ± 187	2,280 ± 159	0.027

Values are expressed as mean RGCs/mm^2^ ± 1 SEM. N = 6 for Sham and N = 11 for Elevated ICP. A t-test was performed for all comparisons. Tuj1 = anti-beta III tubulin; RBPMS = RNA binding protein with multiple splicing; ICP = intracranial pressure.

**Figure 3:**
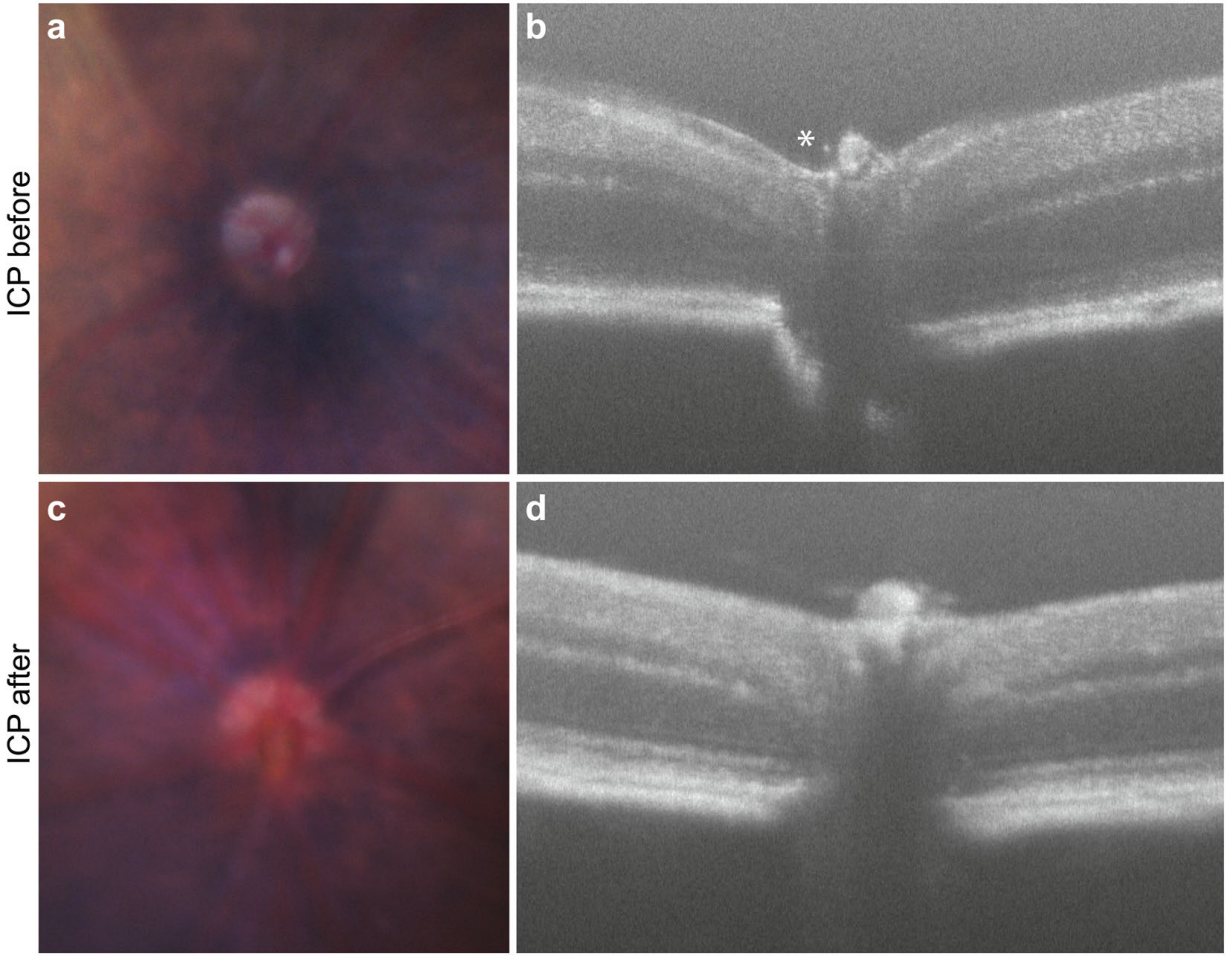
ICP elevation causes optic nerve swelling, hyperemia, and loss of physiologic cupping. Representative fundus and SD-OCT images taken from the same animal before and after 2 weeks of ICP elevation. Fundus photos are cropped to show the region including the optic nerve that is reflected in the corresponding OCT scans. ICP before: (**a**) Fundus photography shows a representative optic disc with clear margins, normal coloration, and surrounding blood vessels of normal caliber; (**b**) SD-OCT shows physiological cupping (asterisk). ICP after: (**c**) Fundus photography shows the same optic disc, now with blurred margins, hyperemic discoloration, and engorged surrounding blood vessels; (**d**) SD-OCT shows a loss of physiological cupping and anterior displacement of the optic disc.

Finally, we used *in vivo* SD-OCT and fundus photography to assess changes to retinal structure in the animals after chronic ICP elevation. Papilledema, loss of physiologic cupping, and engorgement of the optic nerve head and surrounding blood vessels were all detectable with the combination of SD-OCT and fundus photography, suggesting that this model displays critical features of human diseases of elevated ICP (Fig. 3)^1,12–14^. The thickness of each layer of the retina was also determined with SD-OCT and after 2 weeks of ICP elevation, only the thickness of the GCL was found to be statistically reduced, further suggesting that the anatomic impact of elevated ICP is seen primarily in the GCL (Table 2).

**Table 2:** Thickness of retinal layers.

	**Thickness**					
	GCL	IPL	INL	OPL	ONL	Total
Sham before	19.86 ± 0.95	46.33 ± 1.38	22.13 ± 1.19	16.97 ± 1.14	69.27 ± 2.65	174.58 ± 4.26
Sham after	20.90 ± 1.36	46.32 ± 1.50	20.93 ± 1.50	15.93 ± 0.91	65.52 ± 2.32	169.60 ± 4.42
ICP before	21.88 ± 1.31	46.06 ± 1.89	19.63 ± 1.10	16.74 ± 1.12	59.02 ± 2.23	163.33 ± 3.00
ICP after	**18.97** ± **0.87**	50.35 ± 2.41	19.64 ± 1.53	19.12 ± 1.03	56.45 ± 2.44	164.54 ± 3.48

Thickness values are expressed as mean µM ± SEM. N = 6 eyes in each group. For each eye, SD-OCT was used to calculate the thickness of each retinal layer at the start of the experiment (Sham before and ICP before). This was compared to the thickness of each layer in the same eye after 2 weeks (Sham after and ICP after) with a t-test. The only difference detected in any retinal layer was a reduction in the thickness of the GCL after 2 weeks of ICP elevation (bold, p = 0.037). SD-OCT = Spectral domain optical coherence tomography; ICP = intracranial pressure; GCL = retinal ganglion cell layer; IPL = inner plexiform layer; INL = inner nuclear layer; OPL = outer plexiform layer; ONL = outer nuclear layer.

### Sustained ICP elevation causes increased HIF-1α expression

**Figure 4:**
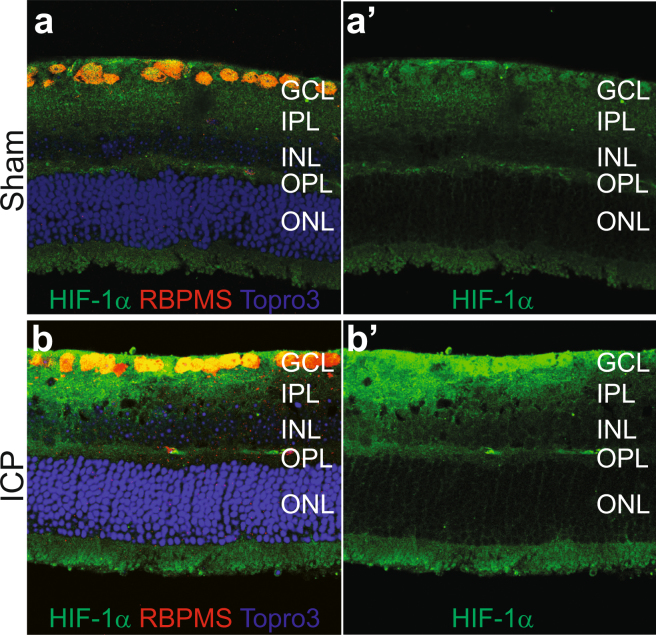
ICP elevation causes increased HIF-1α expression. Representative retinal cross section images from Sham (**a** and **a’**) and ICP (**b** and **b’**) eyes stained for HIF-1α (green), RBPMS (RGCs, red), and Topro3 (nuclei, blue). HIF-1α expression is preferentially increased in the GCL after 2 weeks of ICP elevation (compare **b’** to **a’**; see Table 3). GCL = retinal ganglion cell layer; IPL = inner plexiform layer; INL = inner nuclear layer; OPL = outer plexiform layer; ONL = outer nuclear layer.

These data suggest a predominantly RGC- and RGC-axon based phenotype following ICP elevation. Many theories have been presented in humans to explain optic nerve and retinal injury in the setting of elevated ICP and there is considerable evidence for secondary blood flow abnormalities at the level of the optic nerve head^12^. Such blood flow abnormalities result in perfusion abnormalities which produce secondary hypoxia and oxidative stress in the retina. The expression of HIF-1α protein is increased under conditions of ischemic injury and retinal hypoxia and RGCs have been shown in several disease states to have abnormal levels of HIF-1α and its downstream targets^45^. We therefore assessed retinal expression of HIF-1α following 2 weeks of ICP elevation. When compared to controls, retinal HIF-1α expression was increased, and semi-quantitative analysis showed that this increase was preferential to the GCL (Fig. 4, Table 3).

**Table 3:** Relative HIF-1α intensity in each retinal layer.

	GCL	IPL	INL	OPL	ONL
Sham	1 ± 0.17	1 ± 0.16	1 ± 0.15	1 ± 0.27	1 ± 0.19
Elevated ICP	**2.21** ± **0.37**	1.19 ± 0.09	1.32 ± 0.29	1.26 ± 0.3	2.09 ± 0.86

Normalized intensity is expressed as mean ± SEM. N = 4 sections from 4 eyes in each group. After 2 weeks, Sham and ICP elevation eyes were assessed with antibody to HIF-1α and the mean normalized staining intensity compared with a t-test. The only statistically significant difference detected in any retinal layer was in the GCL (bold, p = 0.042). HIF = hypoxia-inducible factor; ICP = intracranial pressure; GCL = retinal ganglion cell layer; IPL = inner plexiform layer; INL = inner nuclear layer; OPL = outer plexiform layer; ONL = outer nuclear layer.

## Discussion

This manuscript reports improvements to a recently described method of controlled ICP elevation in mice^27^. Specifically, we have adapted our original model for longer duration study at less severe levels of ICP elevation in a commonly used pigmented mouse strain. These changes allowed us to study the impact of elevated ICP on the visual system in much more detail via a combination of electrophysiologic, behavioral, and both *in vivo* and post-mortem anatomic techniques. With this improved approach, our data demonstrate that moderate but sustained ICP elevation in C57BL/6 J mice results in RGC and RGC axon-specific injury, as well as increased HIF-1α expression primarily in the GCL. Furthermore, these experiments confirm that papilledema is present in mice with elevated ICP. Together, these phenotypes are highly similar to the key findings of elevated ICP in humans (papilledema, ischemia, RGC thinning, functional deficit, optic nerve injury), and strongly suggest that this model can be used to study human diseases of elevated intracranial pressure such as IIH and SANS^12–14^. As inducible models of ICP elevation are otherwise lacking, this system is a major improvement over the few other current models of ICP elevation, which either lack the ability to refine ICP to desired parameters of level and duration or are acute in nature^16–18,20^.

While not explored in this manuscript, it is likely that ICP elevation can be maintained in animals beyond the 2 week time point as ICP readings generated by the experimental system are stable for at least 3 weeks and the condition is well-tolerated in most mice^20^. Furthermore, the co-manipulation of ICP and other physiologic parameters is likely possible. For instance, since IOP is not impacted by the elevation of ICP in this system, an experimental model of IOP elevation could be employed concurrently^20^. Other parameters, such as blood pressure, should be equally amenable to concurrent manipulation.

There is a growing body of evidence that the relationship of ICP and IOP at the optic nerve head is critical to optic nerve health. Specifically, an imbalance of pressure in either direction results in a series of characteristic optic nerve changes. This imbalance can occur primarily in four ways: 1) ICP is elevated but IOP is unchanged (IIH, SANS, this manuscript); 2) ICP is unchanged but IOP is reduced (hypotony); 3) IOP is elevated but ICP is unchanged (high tension or secondary glaucoma); and 4) IOP is unchanged but ICP is reduced (normal tension glaucoma). Studies in both animals and humans suggest that in 1 and 2, ICP > IOP, and the result is optic nerve edema, restriction of axoplasmic flow, venous stasis, vascular leakage, and optic nerve head ischemia, whereas in 3 and 4, IOP > ICP, and the result is restriction of axoplasmic flow, optic disc hemorrhage, optic nerve cupping, and optic nerve head ischemia^12,46,47^. Relative optic nerve ischemia is a common endpoint of these imbalances, and likely contributes to progressive RGC axon and optic nerve injury and secondary RGC soma loss. However, it is not entirely clear if the ischemia which occurs in these imbalances is specific to RGCs and their axons or also impacts other regions of the retina. Our data show that, under conditions of elevated ICP, HIF-1α expression is preferentially increased in the GCL, suggesting that an ischemic injury has occurred. While the experiments in this manuscript do not test the hypothesis that hypoxia or ischemia is the cause of RGC death and dysfunction, it is intriguing that there is also evidence for HIF-1α upregulation in the RGCs of glaucoma patients^48^. Furthermore, HIF-1α regulates a number of downstream targets that are altered in the presence of hypoxia, many of which are sufficient to protect RGCs from various forms of injury, and at least one of which is able to protect RGCs from IOP-related injury^49^. Patterns of retinal HIF-1α expression has not been reported in conditions of ICP elevation, so the findings in this manuscript may point to a common injury pathway for both ICP- and IOP-related injury. While it is not clear if the primary HIF-1α-dependent pathways are altered in this model or in other models of ICP-IOP imbalance, this is an area that is appropriate for further study and has the potential to identify pathways that are critical to the regulation of ICP-IOP gradient injury. This model, used in conjunction with established mouse models of IOP modulation^37,50,51^, is well-suited to address these mechanistic considerations.

The findings of papilledema, RGC injury, and HIF-1α upregulation in this manuscript also suggest that a common vascular component of optic nerve injury during ICP elevation is likely preserved in mice. That they are present despite several key anatomic differences between the mouse and primate optic nerve head including the absence of a collagenous lamina and presence of an analogous astrocyte network in mice is all the more telling, as it suggests that the absence of a collagenous lamina in mice does not appear to preclude ICP-IOP relationships that are similar to those seen in humans^52,53^. This is a critical finding as it provides additional support for the use of mice to study human disease of the optic nerve and optic nerve head. Given the ease of experimental manipulation in mice, this represents an opportunity to study important ICP-IOP relationships that result in blinding human disease.

ICP elevation in this study resulted in a reduction of both total axon count and RGC soma count, and the percent reduction for each was approximately the same (33% for axons, between 22% and 35% depending on marker and region for RGCs; Fig. 2 and Table 1), suggesting that the magnitude of RGC loss was similar at the axon and RGC soma level. We observed several examples of non-neuronal injury in the optic nerves of animals exposed to elevated ICP, yet did not see non-RGC cell loss in the retina. Mechanistically, we would therefore anticipate injury at the axon level first, followed by secondary RGC loss, yet the experiments performed in this study do not allow for the clear demonstration of such a timeline. While our previous work at extreme levels of ICP elevation was supportive of such a mechanism, additional experiments looking at additional intermediate time points or a range of ICP (and IOP) levels will be needed to fully make this determination^27^.

## Methods

### Experimental Animals

All protocols and procedures were approved by the Institutional Animal Care and Use Committee of Baylor College of Medicine and were conducted in accordance with the United States Public Health Service’s Policy on Humane Care and the Use of Laboratory Animals. 12-week-old, C57BL/6 J mice were obtained from Jackson Labs (strain 000664) and maintained according to a standard 12hr light and dark cycle. After ICP elevation, mice were housed in a dedicated mouse satellite facility. Mice were housed communally until probe implantation (see below) and then housed singly to prevent instrumentation disruption.

### Animal surgery and ICP elevation

36 (thirty-six) C57BL/6 J mice were used in this study; 19 underwent probe implantation surgery without infusion of artificial CSF (ACSF; Sham) and 17 underwent probe implantation with infusion of ACSF (ICP). Animals were surgically prepared as described previously with modifications^27^. Animals were brought to the surgical plane using a combination anesthetic (ketamine 80 mg/kg, xylazine 16 mg/kg, and acepromazine 1.2 mg/kg) administered intraperitoneally. Subsequently, a 1 cm midline incision was made to expose the bony surface of the skull. Two 1 mm holes were drilled on both sides of skull positioned 1 mm lateral and 1 mm posterior to bregma, and the dura was nicked with a 30-gauge needle to ensure an egress of cerebrospinal fluid (CSF). A customized infusion cannula made from a 22-gauge needle surrounded with nylon screw and containing a central 0.5 mm tunnel (C212SGN, Plastics1) was inserted into one of the holes. A custom, stainless-steel screw with a 0.5 mm central tunnel (C212SG, Plastics1) was inserted into the other hole and used to house the tip of the pressure-monitoring transmitter (PA-C10, Data Sciences International). Both the cannula and screw were anchored to the skull using Durelon Carboxylate Luting Cement (3 M). After solidification of the cement, a 0.5 cm transverse incision was made 2–3 mm posterior to the base of the skull and a pocket made by blunt dissection subcutaneously over the back. The PA-C10 transmitter was placed in this pocket and probe tip passed subdermally and anteriorly into the hole of the stainless-steel screw and into the subarachnoid space. An airtight interface was maintained by applying silicone caulk to the cannula, screw, and anterior-most segment of the PA-C10. For mice that underwent ICP elevation, the infusion cannula was attached to a sterile bottle filled with sterile artificial CSF (ACSF; 124 mM NaCl, 2.5 mM KCl, 2.0 mM MgSO_4_, 1.25 mM KH_2_PO_4_, 26 mM NaHCO_3_, 10 mM glucose, 4 mM sucrose, 2.5 mM CaCl_2_). A slow infusion of ACSF into brain driven by gravity maintained elevation of ICP throughout the study and was titrated to the desired level at the start of the experiment. Sham mice had the infusion cannula sealed off to ambient air by attaching a small length of PE50 tubing with one end heat sealed. In both groups, ICP data were collected wirelessly by the PhysioTel Small Animal Telemetry system for about 1 hour several times a week and data were processed using Ponemah Software 6.11 software (Data Sciences International). All animals were treated with extended release Meloxicam (72 hours) at the time of surgery and then as needed when signs of pain were observed. Breeder chow was provided to all animals as there were some examples of slight weight loss in the later stages of earlier experiments. In the uncommon cases where animals showed obvious signs of distress despite treatment for pain, they were promptly removed from the study and euthanized. Animals were housed singly in a custom caging system designed with input and approval from the veterinary staff.

### Measurement of intraocular pressure (IOP) and optokinetic responses (OKR)

For all animals, IOP was measured with a rebound tonometer (iCare, Tonolab) before surgery and then once each week after the surgery according to standard lab procedure^37^. Mice were anesthetized with inhaled isoflurane for all measurements.

Contrast sensitivity of living mice was measured with an established, custom OKR-based technique^35,54^. Following at least 2 hours of dark adaptation, baseline photopic and scotopic contrast sensitivities were tested on the day after skull surgery, just prior to ICP elevation. Photopic and scotopic OKR experiments were repeated after 2 weeks of ICP elevation or sham treatment.

### Recording of the Electroretinogram (ERG)

ERGs were recorded as previously described with minor modifications^37,55^. In brief, the mice were dark-adapted for at least 2 hours before examination and all the procedures were prepared under dim red light. Mice were anesthetized as above and kept warm with a recirculating heating blanket. A drop of 0.5% proparacaine hydrochloride and a drop of 1% tropicamide were applied to both eyes before all experiments. Scotopic flashes were generated with cyan light emitting diodes calibrated with a photometer. A 0.5 ms light stimulus of 500 nm was used for all flashes. Positive scotopic threshold response (pSTR) measurements were obtained at three levels of stimuli ranging from in log intensity from −4.68 to −3.80 log scot cd*s/m^2^, which are clearly independent from the b-wave which was measured at six levels of stimuli from −2.51 to 0.16 log scot cd*s/m^2^ ^36^. Filtered signals within 0.1 to 1000 Hz were amplified using a P122 amplifier (Grass Instruments, West Warwick, RI). Data were digitized at a sampling rate of 10,000 Hz and further analyzed with custom software written in MATLAB (MathWorks, Natick, MA). The pSTR amplitudes were plotted against the intensity of stimuli and the slope of linear regression was calculated. Amplitude mean value and slope of the linear regression of amplitude growth were then used for statistical comparison.

### Electron microscopy and optic nerve analysis

Eyeballs and optic nerves were removed according to established protocols^27,37,56^. After separation from the eyeball and fixation in 3% glutaraldehyde for up to 72 hours, optic nerves were then washed, post-fixed, dehydrated, embedded in plastic molds, processed, sectioned, stained, and enhanced as described^27^. Images were obtained with a Zeiss EM902 transmission electron microscope (TEM; Carl Zeiss, Germany). Two central regions of the nerve, eight peripheral regions equidistant to the center and circumference of the nerve were imaged at 3000 × magnification and captured with an AMT V602 digital camera. The identity of these regions was masked and myelinated axons counted semi-automatically for all ten regions by two investigators with assistance from a Trainable Weka Segmentation plugin of Fiji ImageJ (National Institutes of Health, Bethesda, MD) to identify the myelinated axons walls. Axon numbers were counted using the “Analyze Particles” function of Fiji ImageJ with the following parameters: size = 0.01 µm^2^-infinity; circularity = 0.4–1; which were empirically determined and validated with a set of 10 optic nerves (Pearson correlation comparing automated to manual counting = 0.84). The axon density was then converted to axons per optic nerve^27,57,58^. The area of each counted region was approximately 625 μm^2^ and the ten counted regions sampled about 5–6% of the total optic nerve cross sectional area.

### Confocal microscopy

Retinas were isolated using established protocols, fixed with 4% paraformaldehyde for one hour, and blocked with 10% donkey serum overnight^37,55^. Fixed retina tissue was then used for flat-mounts or further dissected into 40–50 mm thick slices^59^. Flat-mount retinas and sliced sections were incubated in primary antibodies in the presence of 3% donkey serum-PBS for 3 to 5 days at 4 °C. Primary antibodies used for the study include: mouse anti-beta-III-tubulin (Tuj1; 1:250, Biolegend, Emeryville CA), rabbit anti-RNA binding protein with multiple splicing (RBPMS; 1:250, PhosphoSolutions, Aurora CO), and mouse anti-Hypoxia-inducible factor 1-alpha (HIF-1α; 1:200, Abcam, Cambridge MA). Visualization was achieved with Alexa Fluor488 conjugated donkey anti-mouse IgG (1:300, Molecular Probes, Eugene OR) and Cy3 conjugated donkey anti-rabbit IgG (1:300, Jackson Lab, West Grove PA) accompanied with Topro3 Iodine (Molecular Probes, Eugene OR) for nuclei staining.

Images for RGC counting were acquired with a laser confocal microscope at 40 × magnification (LSM 510; Carl Zeiss) at the center of GCL. Specific regions of the central and peripheral retina were sampled as previously described^37^. Both Tuj1 and RBPMS positive cells were counted manually from all eight regions by two masked investigators, assisted by ImageJ software. Cell counts were then converted into RGC density (RGCs/mm^2^). For HIF-1α studies in cross section, all retina slices were stored in PBS with 0.1% NaN_3_ until immunostaining, then processed under the same conditions and imaged with same confocal settings. Images were segmented to different retinal layers based on nuclear staining and assisted by ImageJ. The mean intensity of HIF-1α signal from sham animals in each retinal layer was set at a value of 1 and the signal from elevated ICP animals was normalized to this value for comparison.

### Optical coherence tomography (OCT)

OCT imaging was performed with either the Envisu SD-OCT (Spectral Domain OCT, Bioptigen, Research Triangle Park, NC) or Micron IV (Phoenix Research Labs, Pleasanton, CA) systems. Mice were anesthetized and then placed on a mount specifically designed for OCT imaging. A drop of 0.5% proparacaine used for anesthetic and 2.5% phenylephrine along with 1% tropicamide were used for pupil dilation. Fundus images were taken to include the optic disc and its surrounding area. A sequence of repeated OCT scan (100 B-scans consisting of 1000 A-scans) was conducted, centered at the optic disc of each eye. Measurements were restricted to an 0.8 mm × 0.8 mm area surrounding the optic disc. OCT sampling was repeated five times and images were registered and averaged to create a high signal-to-noise-ratio image for each animal. Manual segmentation was performed off line and used to measure the thickness of the central retina (200 mm away from optic disc). Experiments were conducted immediately after surgery to implant the ICP probe and again after 2 weeks of ICP elevation.

### Statistical analysis

Statistical analysis was performed in either Excel 2016 (Microsoft, Redmond, WA) or GraphPad Prism 6.0 (GraphPad Software, La Jolla, CA). A student t-test was used to compare the difference between ICP levels, OCT measurements, ERG amplitudes and slope, axon counts, RGC counts, and HIF-1α expression levels. An ANOVA was used for the analysis of OKR results. A cutoff p value of 0.05 was used in all cases.

### Data availability

All data generated or analyzed during this study are included in the published article.

## Electronic supplementary material

Supplementary Information
