# Author Correction: Characterization of Retinal Ganglion Cell and Optic Nerve Phenotypes Caused by Sustained Intracranial Pressure Elevation in Mice

**DOI:** 10.1038/s41598-019-46779-4

**Published:** 2019-10-08

**Authors:** Guofu Shen, Schuyler Link, Sandeep Kumar, Derek M. Nusbaum, Dennis Y. Tse, Yingbin Fu, Samuel M. Wu, Benjamin J. Frankfort

**Affiliations:** 1 Department of Ophthalmology, Baylor College of Medicine, Houston, TX USA; 2 Department of Neuroscience, Baylor College of Medicine, Houston, TX USA; 3 School of Optometry, The Hong Kong Polytechnic University, Hong Kong, Hong Kong

Correction to: *Scientific Reports* 10.1038/s41598-018-21254-8, published online 12 February 2018

The Acknowledgements section in this Article is incomplete.

“This research was supported by the NIH (R01 EY025601 to B.J.F., R01 EY019908 to S.M.W., R01 EY022901 to Y.F., and P30 EY002520 to S.M.W.), with additional funding from Research to Prevent Blindness (Baylor College of Medicine), the Retina Research Foundation (S.M.W. and B.J.F.) and Hong Kong Polytechnic University grant # G-UA7J (D.Y.T.). We thank Dr. Yong Park for critically reading the manuscript.”

should read:

“This research was supported by the NIH (R01 EY025601 to B.J.F., R01 EY019908 to S.M.W., and P30 EY002520 to S.M.W.), with additional funding from Research to Prevent Blindness (Baylor College of Medicine), the Retina Research Foundation (S.M.W. and B.J.F.) and Hong Kong Polytechnic University grant # G-UA7J (D.Y.T.). We thank Dr. Yong Park for critically reading the manuscript.”

