# Supplementary material for: Characterization of Retinal Ganglion Cell and Optic Nerve Phenotypes Caused by Sustained Intracranial Pressure Elevation in Mice
Source: Sci Rep. 2018 Feb 12;8:2856. doi: 10.1038/s41598-018-21254-8 (PMC5809383; doi:10.1038/s41598-018-21254-8)
Supplement: Supplementary file 1 — Supplementary Information [file 41598_2018_21254_MOESM1_ESM.pdf]

Title: Characterization of Retinal Ganglion Cell and Optic Nerve Phenotypes Caused by Sustained Intracranial Pressure Elevation in Mice

Guofu Shen, PhD,[1] Schuyler Link,[1] Sandeep Kumar, PhD,[1] Derek M. Nusbaum, MD, PhD,[1,2] Dennis Y. Tse, PhD,[1,3] Yingbin Fu, PhD,[1,2] Samuel M. Wu, PhD,[1,2] and Benjamin J. Frankfort, MD, PhD[1,2,*]

[1]Department of Ophthalmology, Baylor College of Medicine, Houston, TX
[2]Department of Neuroscience, Baylor College of Medicine, Houston, TX
[3]School of Optometry, The Hong Kong Polytechnic University, Hong Kong

* Corresponding author:
Benjamin J. Frankfort, MD, PhD
Baylor College of Medicine
Department of Ophthalmology
6565 Fannin St., NC-205, Houston, TX 77030.
benjamin.frankfort@bcm.edu

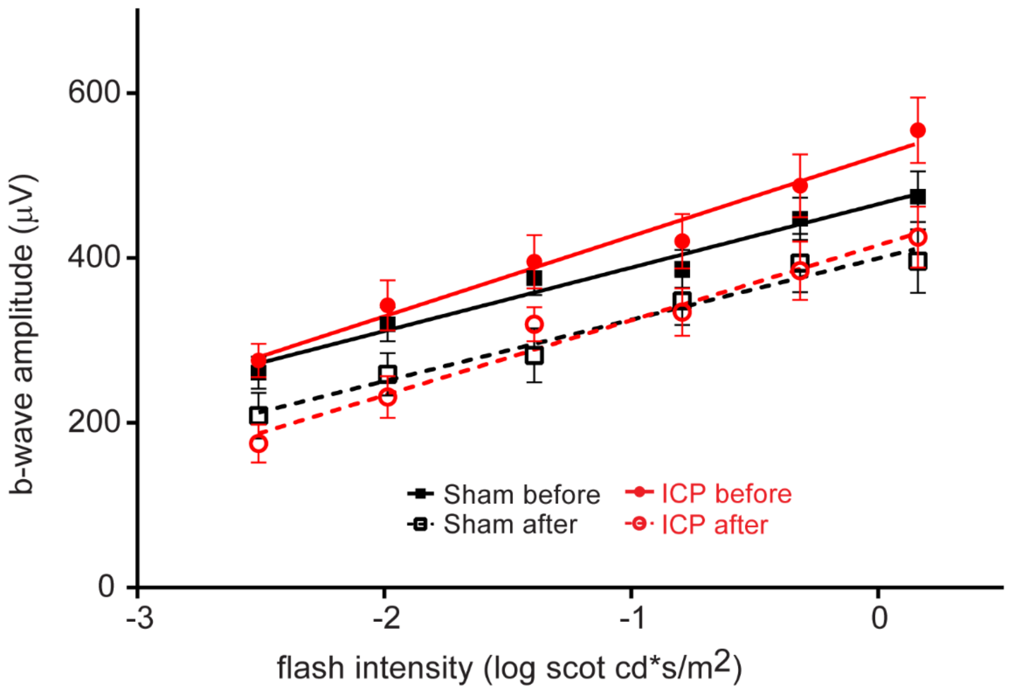

Supplemental Figure S1. b-wave amplitudes before and after ICP elevation. Mean electroretinogram (ERG) b-wave amplitude plotted against the intensity of scotopic flash stimuli for Sham (black) and ICP (red) animals. The best-fit linear regression lines of b-wave amplitude growth are indicated for each condition (red and black solid and dotted lines). The magnitude of the linear regression slope of b-wave growth is equivalent for both Sham before to ICP before and Sham after to ICP after, as well as for intra-group comparisons of Sham before to Sham after and ICP before to ICP after ($p > 0.05$ for all). The mean amplitude of the b-wave decreased in both groups equally after instrumentation. N = 26 eyes for Sham and N = 12 eyes for ICP. Error bar = 1 SEM.

	Sham	Elevated ICP	P
All cells - central	8,272 ± 598	6,539 ± 360	0.018
All cells - peripheral	7,214 ± 479	6,125 ± 320	0.070
Non-RGCs A - central	3,798 ± 268	3,613 ± 148	0.521
Non-RGCs A - peripheral	2,908 ± 227	2,966 ± 113	0.800
Non-RGCs B - central	4,834 ± 452	4,044 ± 207	0.087
Non-RGCs B - peripheral	4,307 ± 324	3,845 ± 188	0.204

Supplemental Table S1. Non-RGC cells in the GCL.

Values are expressed as mean cells/mm^2 ± 1 SEM. N = 6 for Sham and N = 11 for Elevated ICP. A t-test was performed for all comparisons. “All cells” indicates the number of nuclei in the GCL detected by the marker Topro3. Since RGC markers differ in both their specificity and sensitivity, we calculated the number of non-RGCs in two ways: 1) as the the difference of Topro3 cells and Tuj1 (Non-RGCs A) and 2) as the difference of Topro3 cells and RBPMS cells (Non-RGCs B). In all cases, there was no statistically significant difference in non-RGC cells within the GCL between Elevated ICP and Sham retinas. All cells are statistically reduced in Elevated ICP retinas in some instances and this is driven by RGC loss. ICP = intracranial pressure.
